# Podophyllotoxin-Loaded Nanostructured Lipid Carriers for Skin Targeting: In Vitro and In Vivo Studies

**DOI:** 10.3390/molecules21111549

**Published:** 2016-11-17

**Authors:** Jihui Zhao, Xianghua Piao, Xiaoqin Shi, Aiyong Si, Yongtai Zhang, Nianping Feng

**Affiliations:** School of Pharmacy, Shanghai University of Traditional Chinese Medicine, Shanghai 201203, China; zhaojihui07168@163.com (J.Z.); piaoxianghua721@126.com (X.P.); xiaoqin_shi87@163.com (X.S.); giantison@163.com (A.S.); analysisdrug@126.com (Y.Z.)

**Keywords:** podophyllotoxin, nanostructured lipid carriers, skin targeting efficiency

## Abstract

Nanostructured lipid carriers (NLC) exhibit high skin targeting efficiency and good safety. They are promising vehicles for topical drug delivery. This study aims to increase the skin distribution of podophyllotoxin (POD) by incorporating it into NLCs. Two kinds of POD-loaded NLCs (POD-NLCs)—POD-NLC_formulation 1_ and POD-NLC_formulation 2_—were prepared and characterized. Their skin targeting efficiencies were compared by conducting in vitro and in vivo experiments. Obviously smaller mean particle size was observed for POD-NLC_formulation 1_ (106 nm) than POD-NLC_formulation 2_ (219 nm), whereas relatively low POD loadings (less than 0.5%) were observed for both POD-NLC_formulation 1_ (0.33%) and POD-NLC_formulation 2_ (0.49%). Significantly higher in vitro and in vivo rat skin deposit amounts of POD (*p* ˂ 0.01) were detected after the topical application of POD-NLC_formulation 1_ compared to POD-NLC_formulation 2_. To visualize the skin distribution behavior of hydrophobic active pharmaceutical ingredients (APIs) when NLCs were used as carriers, POD was replaced with Nile red (NR—a hydrophobic fluorescent probe), and the distribution behavior of NR-NLC_formulation 1_ and NR-NLC_formulation 2_ in rat skin in vivo was observed using confocal laser scanning microscopy (CLSM). Higher fluorescent intensity was observed in rat skin after the topical application of NR-NLC_formulation 1_ than NR-NLC_formulation 2_, suggesting that higher skin targeting efficiency might be obtained when NLCs with smaller mean particle size were used as carriers for hydrophobic APIs. This result was in accordance with those of skin distribution evaluation experiments of POD-NLCs. Skin irritation property of POD-NLC_formulation 1_ was investigated and no irritation was observed in intact or damaged rabbit skin, suggesting it is safe for topical use. Our results validated the safety of NLCs when applied topically. More importantly, mean particle size might be an important parameter for formulation optimization when NLCs are used as carriers for hydrophobic APIs for topical application, considering that their loading is relatively low.

## 1. Introduction

In the treatment of skin diseases, the efficacy of topical drugs depends on their ability to reach the desired site of action (specific skin layers) and remain at the site in an effective concentration for the appropriate time. Drug safety depends critically on the tight control of release rate and target specificity by the delivery vehicle. However, conventional topical formulations, such as solutions, ointments, and gels are characterized by indiscriminate skin targeting properties and rapid drug release. When these vehicles are employed as carriers, topical drugs such as triamcinolone, 5α-dihydrotestosterone, and tretinoin exhibit poor therapeutic effects, as well as mild to severe local and systemic adverse reactions [1,2,3]. The development of novel drug delivery systems that control both target specificity and drug release rates is urgently needed in the dermatological field.

Liposomes were first described by Bangham and co-workers in the 1960s [4]. Liposomes can encapsulate both hydrophilic and lipophilic drugs, and were first used for systemic and then topical drug delivery. When used as topical carriers, liposomes can confer skin targeting and prolonged temporal release properties of the loaded drug. They can also act to reduce topical and systemic adverse reactions, because their small particle size enables close contact with the superficial junctions of corneocyte clusters and furrows between corneocyte islands. The similarities between liposome components and epidermal lipids promote lipid exchange and facilitate penetration of the loaded drug through the stratum corneum to deeper skin layers, where the drug is deposited [5,6]. Liposomes have been used as carriers for many topical drugs; e.g., corticosteroids, retinoids, and local anesthetics [7]. However, the main drawback of liposomes as drug carriers is their poor physical stability—namely, drug leakage during storage.

Solid lipid nanoparticles (SLNs)—the first generation of lipid nanoparticles—are composed of lipids that are solid at room temperature and which are covered by a surfactant shell that stabilizes their dispersion. The first SLNs were developed in the early 1990s. SLNs are considered to have all the advantages of liposomes when used as topical carriers, and can also improve the physical and chemical stability of loaded drugs [8,9]. SLNs have been used as topical carriers for many lipophilic drugs, such as isotretinoin, retinyl palmitate, prednicarbate, tacrolimus, penciclovir, clotrimazole, and antioxidants including lutein, curcuminoids, quercetin, and idebenone [10,11,12,13,14,15,16,17,18,19].

**Figure 1:**
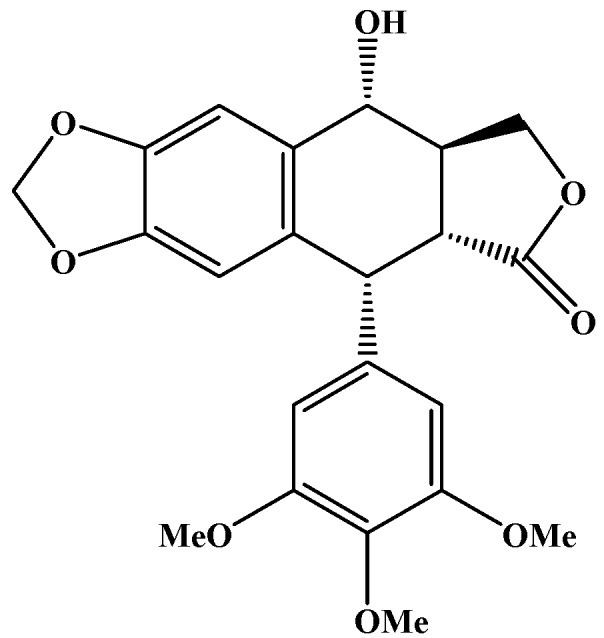
Chemical structure of podophyllotoxin (POD).

Condyloma accuminata (CA) is one of the most common sexually transmitted diseases. Podophyllotoxin (POD, an antimitotic agent), can destroy warts by inducing tissue necrosis and has been recommended by the World Health Organization (WHO) as the first line drug for the treatment of CA [20]. The chemical structure of POD is shown in Figure 1. However, the efficacy of 0.5% POD tincture and cream against CA is considerably compromised by a high recurrence rate (approximately 40%), skin irritation, and possible systemic toxicity [21,22,23].

Chen et al. prepared and compared two 0.15% POD-loaded SLNs with different mean particle sizes [24]. Neither SLN enabled the systemic absorption of POD, and the SLN with the smaller mean particle size exhibited higher epidermal targeting efficiency than a 0.15% POD tincture. These data suggest that SLNs are a promising carrier for the topical delivery of POD. However, the POD content in SLNs is approximately one third of that in the available tinctures or creams, which could compromise its therapeutic efficiency against CA. POD-loaded SLNs may also exhibit a serious physical stability problem; namely, drug expulsion during storage [25].

Nanostructured lipid carriers (NLCs, the second generation of lipid nanoparticles), are mixtures of solid and fluid lipids. The fluid lipid phase is thought to be embedded in the solid lipid matrix or to be localized at the interphase of the solid platelets and the surface layer. The introduction of fluid lipids still allows the lipid nanoparticles to remain in a solid state at room temperature, but the crystal lattice in the lipid particles becomes somewhat imperfect, improving both drug loading capacity and physical stability [26]. NLCs have been used as topical carrier for many drugs, such as acitretin, non-steroidal anti-inflammatory drugs (NSAIDs; ketoprofen, indomethacin), antioxidants (lutein, CoQ10), flubiprofen, clotrimazole, and celecoxib [27,28,29,30,31,32]. These encouraging results suggest that POD-loaded NLCs will exhibit enhanced therapeutic efficiency against CA.

In the present study, POD was loaded into NLCs to improve its skin targeting efficiency. Two kinds of POD-NLCs were prepared and characterized, and their skin targeting efficiencies were compared. A preliminary safety evaluation of POD-NLC exhibiting higher skin targeting efficiency was also conducted.

## 2. Results and Discussion

### 2.1. Mean Particle Size, Polydispersity Index, and Zeta Potential

The mean particle size of POD-NLC_formulation 1_ and POD-NLC_formulation 2_ was 106 nm and 219 nm, respectively. Both preparations have a relatively uniform particle size, as indicated by their small polydispersity indexes (0.211 for POD-NLC_formulation 1_ and 0.221 for POD-NLC_formulation 2_), and have similar zeta potentials (−18.00 mV for POD-NLC_formulation 1_ and −16.4 mV for POD-NLC_formulation 2_).

### 2.2. POD Loading and Entrapment Efficiency

The POD content in both NLCs was relatively low (less than 0.5%), though a slightly higher content of POD was seen in POD-NLC_formulation 2_ (0.49%) compared to POD-NLC_formulation 1_ (0.33%). Both NLCs also had high POD entrapment efficiencies (88.25% for POD-NLC_formulation 1_ and 93.50% for POD-NLC_formulation 2_).

### 2.3. Differential Scanning Calorimetry (DSC)

**Figure 2:**
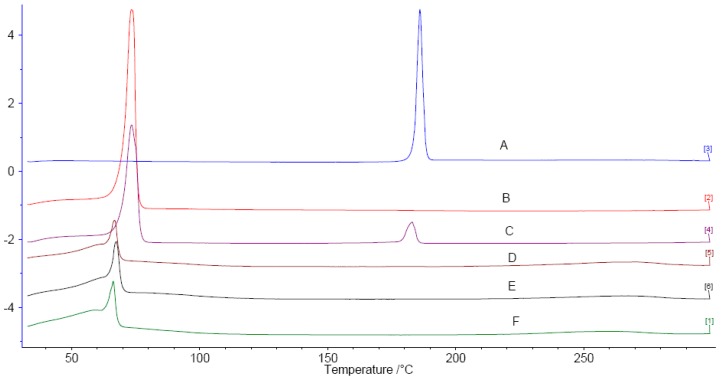
Differential Scanning Calorimetry (DSC) heating curves of podophyllotoxin-loaded nanostructured lipid carriers (POD-NLC)_formulation 1_ and POD-NLC_formulation 2_ from 30 to 300 °C at a heating rate of 10 °C/min. **A**: POD; **B**: Compritol 888 ATO; **C**: Physical mixture of POD and Compritol 888 ATO; **D** to **F**: Lyophilized NLC dispersions—namely, POD-NLC_formulation 1_, POD-NLC_formulation 2_, and Blank-NLC_formulation 1_.

The DSC thermograms for POD, Compritol^®^ 888 ATO, a physical mixture of POD and Compritol^®^ 888 ATO, and lyophilized samples of blank NLC_formulation 1_, POD-NLC_formulation 1_, and POD-NLC_formulation 2_ are shown in Figure 2. Melt peaks for pure POD and Compritol^®^ 888 ATO were observed at 186.0 and 73.5 °C, respectively. The corresponding melt peaks are 182.8 and 73.4 °C in their physical mixture, suggesting that the physical state of the drug and solid lipid were not affected by mixing. The slight difference in the melt temperatures of POD alone and POD in the physical mixture is probably due to measurement error. No melt peak was observed for POD in the thermograms of POD-loaded NLCs, indicating either complete solubilization of POD in the lipid matrix or transformation of POD crystal to an amorphous form that has been dispersed in the lipid matrix [33]. Compared with Compritol^®^ 888 ATO alone, the melt peak for Compritol^®^ 888 ATO was broadened and shifted to a lower temperature in blank and POD-loaded NLCs. The large surface area of nanoparticles and other excipients such as oil and surfactant may act as impurities that affect the melting point of Compritol^®^ 888 ATO [34].

### 2.4. In Vitro Release

**Figure 3:**
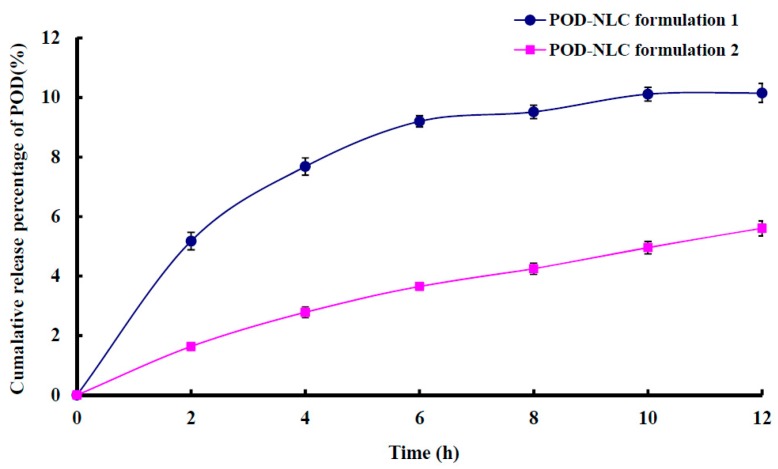
Cumulative in vitro release percentages of POD from POD-NLC_formulation 1_ and POD-NLC_formulation 2_ versus time profiles.

Cumulative in vitro release percentages of POD from NLCs are shown in Figure 3. POD was released slowly from both NLCs, and less than 10% of loaded POD was released over a 12 h period. The cumulative in vitro release percentage of POD was higher in POD-NLC_formulation 1_ than POD-NLC_formulation 2_, possibly because POD-NLC_formulation 1_ has a larger surface area and therefore presents a larger contact area to the release medium.

### 2.5. In Vitro and In Vivo Skin Retention

**Figure 4:**
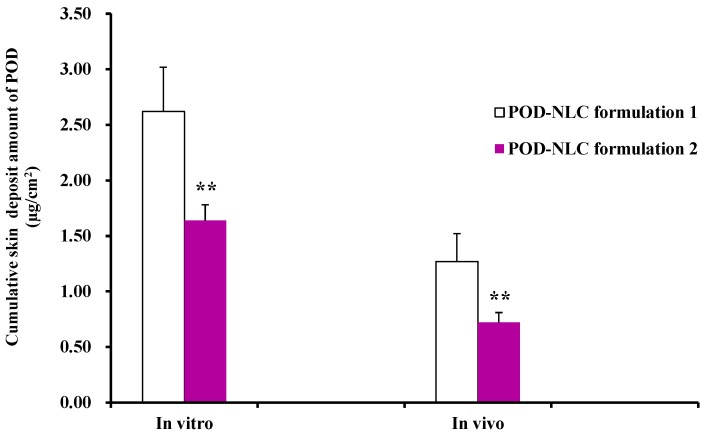
In vitro and in vivo rat skin deposit amounts of POD at 8 h after the topical treatment of POD-NLC_formulation 1_ or POD-NLC_formulation 2_. Note: “**” represents *p* ˂ 0.01 compared with POD-NLC_formulation 1_.

Figure 4 shows the in vitro and in vivo rat skin deposits of POD at 8 h after the application of POD-NLCs. The difference between POD-NLC_formulation 1_ and POD-NLC_formulation 2_ is highly significant (*p* ˂ 0.01), and the skin deposit value for POD-NLC_formulation 1_ is nearly twice that for POD-NLC_formulation 2_. The results suggest that the mean particle size of NLCs has a considerable influence on the skin targeting efficiency of hydrophobic APIs loaded into them. A potential explanation is that lipid particles form an adhesive layer occluding the skin surface after water evaporates from the lipid nanodispersion [5]. As a consequence, hydration of the stratum corneum may increase and facilitate drug penetration into skin. The highest occlusivity will be achieved by NLCs containing the smallest particles [8].

### 2.6. Confocal Laser Scanning Microscopy (CLSM)

To visualize the skin distribution behavior of hydrophobic active pharmaceutical ingredients (APIs) when NLCs were used as carriers, POD was replaced with Nile red (NR—a hydrophobic fluorescent probe), and the distribution behavior of NR-NLC_formulation 1_ and NR-NLC_formulation 2_ in rat skin in vivo was observed using confocal laser scanning microscopy (CLSM). The mean particle sizes of NR-NLC_formulation 1_ and NR-NLC_formulation 2_ were 120 nm and 219 nm, respectively. These results indicate that replacing POD with NR does not influence the particle size of the NLCs.

**Figure 5:**
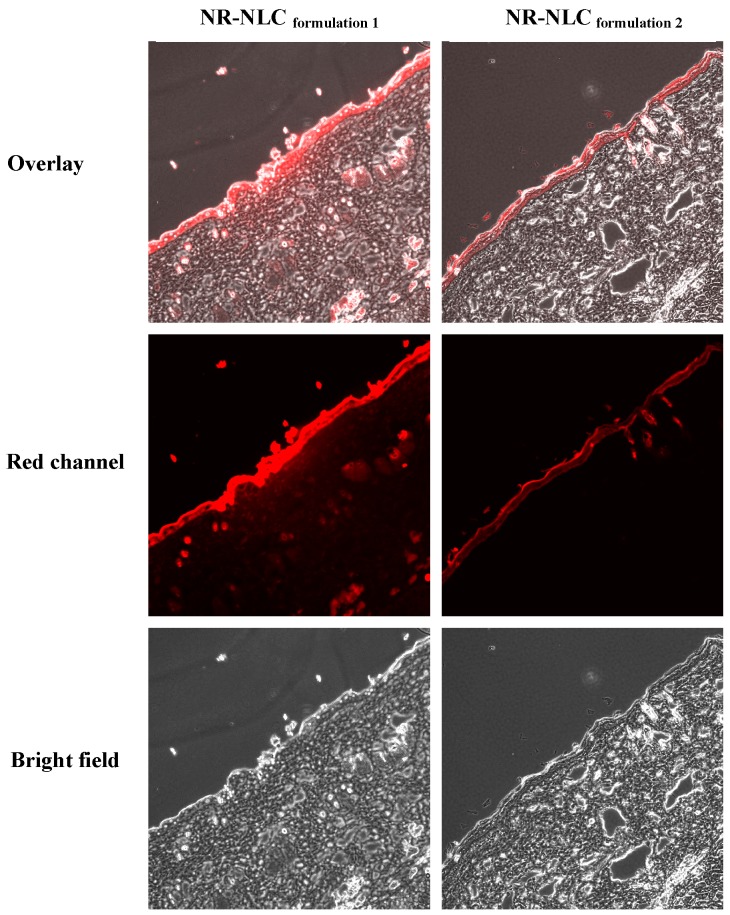
Confocal laser scanning microscopy (CLSM) images of vertical slices (10 μm) of rat skin, 4 h after the administration of Nile red-loaded NLC (NR-NLC)_formulation 1_ (109.7 nm) and NR-NLC_formulation 2_ (219.4 nm).

CLSM images of vertical slices of rat skin obtained 4 h after the application of NR-NLCs are shown in Figure 5. For both NLCs, NR is distributed primarily in the epidermal layer of the skin, indicative of high skin targeting efficiency. Our results are consistent with those reported previously [35,36,37]. By visual inspection, the fluorescence intensity of NR appears to be higher in the epidermal layer after the application of NR-NLC_formulation 1_ than that of NR-NLC_formulation 2_, suggesting that the NLC with the smaller mean particle size has higher epidermal targeting efficiency. This result is in accordance with results from the in vitro and in vivo skin retention experiments, and supports the conclusion that drugs loaded in NLCs are mainly targeted to the epidermal layer.

### 2.7. Skin Irritation Evaluation

**Figure 6:**
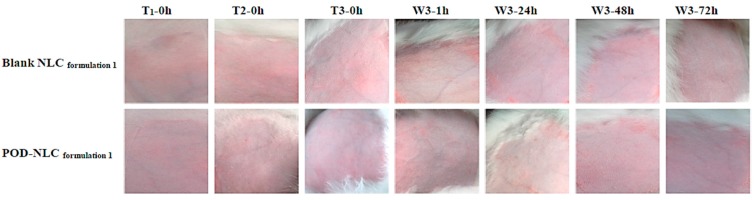
Images of intact rabbit skin before and after three times of topical administration of POD-NLC_formulation 1_ or blank NLC_formulation 1_. Notes: T_1_-0 h, T_2_-0 h and T_3_-0 h represent immediately before the first, second, and third times of administration; W3-1 h, W3-24 h, W3-48 h, and W3-72 h represent 1, 24, 48, and 72 h after the washing off of the residual of the formulation after the last time of administration.

**Figure 7:**
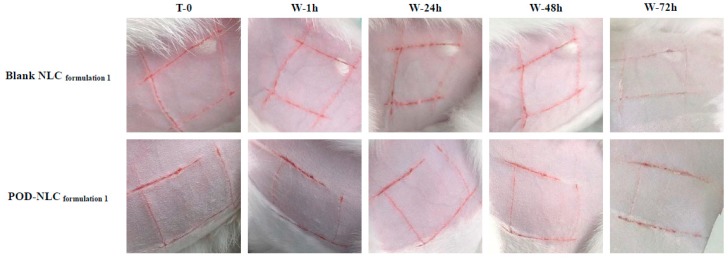
Images of damaged rabbit skin before and after the single-time topical administration of POD-NLC_formulation 1_ or blank NLC_formulation 1_. Notes: T_0_ represents immediately before the administration; W-1 h, W-24 h, W-48 h, and W-72 h represent 1, 24, 48, and 72 h after the washing off of the residual of the formulation.

Representative images of intact or damaged rabbit skin before and after NLC treatment are shown in Figure 6 and Figure 7. No irritation was observed after multiple-dose administration of POD-NLC_formulation 1_ or Blank-NLC_formulation 1_ to intact skin. A single dose of these agents also failed to irritate skin damaged by wounding. These promising results suggest that POD-NLC_formulation 1_ can safely deliver POD.

## 3. Materials and Methods

### 3.1. Reagents

Podophyllotoxin (POD) was obtained from HUAHAI Pharmaceutical Co., Ltd., Fujian, China. Cremophor^®^ RH 40 (Polyoxyl 40 hydrogenated castor oil) was purchased from BASF SE, Ludwigshafen, Germany. Compritol^®^ 888 ATO (Glyceryl behenate) and Labrasol^®^ (Caprylocaproyl macrogolglycerides) were provided by Gattefossè, Saint Priest, France. Soybean phosphatidylcholine (SPC) was purchased from Shanghai Taiwei Pharmaceutical Company Limited, Shanghai, China. Other chemicals were of HPLC or analytical grade.

### 3.2. Preparation of Podophyllotoxin-Loaded Nanostructured Lipid Carriers

Podophyllotoxin-loaded nanostructured lipid carriers were prepared by hot high pressure homogenization. Briefly, Compritol^®^ 888 ATO and Labrasol^®^ were melted at 80 °C in a water bath. POD was dissolved in 5 mL dichloromethane, and the melted lipids were added. The organic solvent was removed by magnetic stirring at 800 rpm for 15 min at room temperature, and the oil phase was then collected. To generate the aqueous phase, Cremophor RH40^®^ and SPC were dissolved in 100 mL of purified water at 80 °C in a water bath, using magnetic stirring at 500 rpm for 2 min. The aqueous phase was added to the oil phase slowly using magnetic stirring at 500 rpm. The mixture was then stirred at 8000 rpm for 10 min to obtain the pre-emulsion. Finally, the mixture was homogenized to obtain the dispersion of POD-NLC using nine cycles of pressurization at 600 bar and cooling to 5 °C. The formulations of POD-NLCs are shown in Table 1.

### 3.3. Mean Particle Size, Polydispersity Index, and Zeta Potential

The particle size distribution (mean particle size and polydispersity index) and zeta potential analysis of POD-NLCs were performed using a Malvern Nano ZS90 Zetasizer (Malvern Instruments Ltd., Worcestershire, UK). Samples were diluted 50-fold in purified water before measurement.

### 3.4. POD Loading and Entrapment Efficiency

The entrapment efficiencies (EE) of POD in POD-NLCs were measured using centrifugal ultrafiltration as previously described [38]. Briefly, a 0.5 mL sample of NLC dispersion was centrifuged at 8000 rpm for 20 min in a centrifugal ultrafiltration tube (Molecular weight cutoff 10 kDa), and the filtrate was subjected to HPLC to determine free POD content (W_f_). An identical volume of NLC dispersion was dissolved in methanol and subjected to HPLC to determine total POD content (W_t_). EE and POD loading were calculated as follows:

$$\mathrm{Entrapment}\;\mathrm{efficiency}\;\left(\mathrm{EE}\right)=\frac{\left(W_{t}-W_{f}\right)}{W_{t}}\times 100\%$$

$$\mathrm{Loading}=\frac{\left(W_{t}-W_{f}\right)}{W_{n}}\times 100\%$$

where W_f_ is the amount of free POD, W_t_ is the amount of total POD present in 0.5 mL of NLC dispersion, and W_n_ is the volume of NLC dispersion (0.5 mL).

### 3.5. Differential Scanning Calorimetry (DSC)

Thermal analysis of POD, Compritol^®^ 888 ATO, the physical mixture of POD with Compritol^®^ 888 ATO, and lyophilized samples of blank NLC_formulation 1_, POD-NLC_formulation1_ and POD-NLC_formulation 2_ was performed using a NETZSCH 240 F1 Differential Scanning Calorimeter (NETZSCH Instrument Ltd., Selb/Bavaria, Germany). DSC was performed using 3 to 5 mg samples. Heating curves were recorded in the range of 30 to 300 °C at a heating rate of 10 °C/min under inert atmosphere (N_2_, 8 mL/min).

### 3.6. In Vitro Release

The release of POD-NLCs was examined in vitro using a Franz diffusion cell system. The effective penetration area and receptor compartment volume were 2.0 cm^2^ and 12.5 cm^3^, respectively. Nitrocellulose membrane filter (pore size 0.1 μm, Sartorius AG, Germany) were used as barriers. Donor compartments contained either 0.25 mL POD-NLC_formulation 1_ or an identical volume of POD-NLC_formulation 2_, and receptor compartments contained normal saline as receptor medium. Receptor medium was maintained at 37 °C in a water bath with magnetic stirring at 300 rpm to ensure the uniform distribution of released POD. Samples of 0.5 mL were withdrawn from the receptor compartments at 0, 2, 4, 6, 8, 10, and 12 h, and replaced with blank medium at the same volume at 37 °C. POD concentration was measured to determine cumulative release percentages by an HPLC method that we reported previously [39].

### 3.7. In Vitro Skin Retention

In vitro skin retention experiments were conducted using the in vitro release protocol, except the nitrocellulose membrane filter was replaced with freshly excised abdominal skin from male Sprague-Dawley rats, with the epidermal surface oriented towards the donor compartment. Eight hours after the application of POD-NLC_formulation 1_ or POD-NLC_formulation 2_, the skins (*n* = 5 for each NLC formulation) were removed and rinsed with blank normal saline at 45 °C. A sample of 1 cm^2^ was taken from each harvested skin, cut into small pieces, and suspended in 0.5 mL normal saline. Each sample was homogenized in an ice bath and centrifuged at 5000 rpm for 10 min. The supernatant (0.2 mL) was extracted for 3 min in 0.6 mL ethyl acetate using a vortex mixer. After centrifugation at 5000 rpm for 10 min, the ethyl acetate layer was recovered and evaporated to dryness under a gentle nitrogen stream. The residue was reconstituted with 0.2 mL of methanol before POD level determination by the same HPLC method mentioned above.

### 3.8. In Vivo Skin Retention

Male Sprague-Dawley rats were anaesthetized with an intraperitoneal injection of urethane (1.5 g/kg). After the abdominal fur was carefully removed with an electric clipper, rats were placed on heating pads maintained at 37 ± 1 °C. Then, 0.28 mL of POD-NLC_formulation 1_ or POD-NLC_formulation 2_ was applied to the abdominal skin (2.3 cm^2^) of each rat. The rats were kept in a dark room during the experiments to minimize POD photodegradation. After 8 h, the animals were sacrificed (*n* = 3 for each NLC formulation) and the treated skin excised. The tissue was wiped clean with a sponge soaked in blank normal saline at 45 °C, and then a sample of 1 cm^2^ was taken from each skin. Skin sample preparation and HPLC conditions were identical to those used in the in vitro experiments described above.

### 3.9. Confocal Laser Scanning Microscopy (CLSM)

To compare differences in skin distribution behavior exhibited by NLCs with different properties, POD was replaced with Nile red (NR, a commonly used fluorescent probe), and NR loaded NLCs were prepared. The characteristics of NR-NLC_formulation 1_ and NR-NLC_formulation 2_ are shown in Table 2. The mean particle size and polydispersity index (PDI) of NR-NLC_formulation 1_ or NR-NLC_formulation 2_ was measured to confirm their similarity to POD-NLC_formulation 1_ or POD-NLC_formulation 2_, respectively.

In vivo rat skin deposit experiments using NR-NLCs (*n* = 3 for each formulation) were conducted following the same procedures for POD-NLCs, except that the rats were sacrificed 4 h after administration instead of 8 h. Excised skin was wiped clean with a sponge soaked in blank normal saline at 45 °C, and then 1 cm samples were embedded in optimal cutting temperature compound (OCT). Three vertical slices (10 μm) of each sample were obtained using a Leica CM1850 cryostat (Leica Inc., Wetzlar, Germany). The distribution of the fluorescent marker was observed using an Olympus FluoView FV10i confocal microscope (Olympus, Tokyo, Japan). Skin slices were examined under both normal and UV (excitation at 559 nm) light, and emission spectra were recorded from 570 to 670 nm. CLSM images in bright-field and red channels were superimposed to visualize NR distribution.

### 3.10. Skin Irritation Evaluation

The potential for POD-NLC_formulation 1_ to irritate normal skin after multiple-dose administration was investigated according to OECD Guideline 404, “Acute Dermal Irritation/Corrosion” [40]. The effects of POD-NLC_formulation 1_ on compromised skin after single-dose administration were also evaluated.

Eight New Zealand rabbits (2.0–2.5 kg) were randomly divided into normal and compromised skin groups (*n* = 4 in each group, half male and half female). A small region on both flanks of each rabbit (approximately 3 × 3 cm) was shaved with an electric clipper. Rabbits in the compromised skin group were wounded in a “#”-shaped pattern on both flanks to induce staxis and were allowed 24 h to recover before administration of POD-NLC_formulation 1_.

In the normal skin group, 0.5 mL of POD-NLC_formulation 1_ or Blank NLC_formulation 1_ was spread evenly onto a gauze patch (approximately 2.5 × 2.5 cm). Patches were applied to the left and right flank of each rabbit and secured with non-irritating elastic bandage. Patches were applied for four hours each day for three days. While the patches were in place, the rabbits were restrained. Treated regions were visually inspected and photographed before patches were applied and after they were removed. When a patch was removed, the area was swabbed with warm normal saline before observations were recorded. Rabbits in the compromised group received only one 4 h treatment with 0.5 mL of POD-NLC_formulation 1_ or Blank NLC_formulation 1_. Before patches were applied, and at 1, 24, 48, and 72 h after patches were removed, the treated areas on both flanks of each rabbit were observed for any evidence of erythema and edema and were photographed.

## 4. Conclusions

It has been widely reported that drugs exhibit high skin targeting efficiency when NLCs are used as vehicles for topical delivery. In this study, POD-loaded NLCs with smaller mean particle size exhibited higher skin targeting efficiency. Mean particle size might be an important parameter for formulation optimization when NLCs are used as carriers for hydrophobic APIs for topical application, considering that their loading is relatively low.

## Figures and Tables

**Table 1:** Formulation of POD-NLCs. PC: phosphatidylcholine.

Drug and Excipients (g/100 mL)	POD-NLC_formulation 1_	POD-NLC_formulation 2_
POD	0.37	0.52
Compritol^®^ 888 ATO	4.30	10.0
Labrasol^®^	0.76	1.8
Cremophor^®^ RH40	5.02	11.7
SPC	2.51	5.85

**Table 2:** Formulations of NR-NLCs.

Drug and Excipients (g/100 mL)	NR-NLC_formulation 1_	NR-NLC_formulation 2_
Nile red	0.005	0.005
Compritol^®^ 888 ATO	3.54	8.2
Labrasol^®^	0.76	1.8
Cremophor^®^ RH40	5.02	11.7
SPC	2.51	5.85
