# Correction: Zhao, J., et al. Podophyllotoxin-Loaded Nanostructured Lipid Carriers for Skin Targeting: In Vitro and In Vivo Studies. *Molecules* 2016, *21*, 1549

**DOI:** 10.3390/molecules21121695

**Published:** 2016-12-09

**Authors:** Jihui Zhao, Xianghua Piao, Xiaoqin Shi, Aiyong Si, Yongtai Zhang, Nianping Feng

**Affiliations:** School of Pharmacy, Shanghai University of Traditional Chinese Medicine, Shanghai 201203, China; zhaojihui07168@163.com (J.Z.); piaoxianghua721@126.com (X.P.); xiaoqin_shi87@163.com (X.S.); giantison@163.com (A.S.); analysisdrug@126.com (Y.Z.); npfeng@hotmail.com (N.F.)

The authors wish to make the following correction to their paper [1]. In Figure 4, Figure 5 and Figure 6, the legend was incorrectly displayed. The corrected legends of Figure 4, Figure 5 and Figure 6 are as follows:

These changes do not affect the scientific results. The manuscript will be updated and the original will remain online on the article webpage. The authors would like to apologize for any inconvenience caused to readers by these changes.

## Figures and Tables

**Figure 4:**
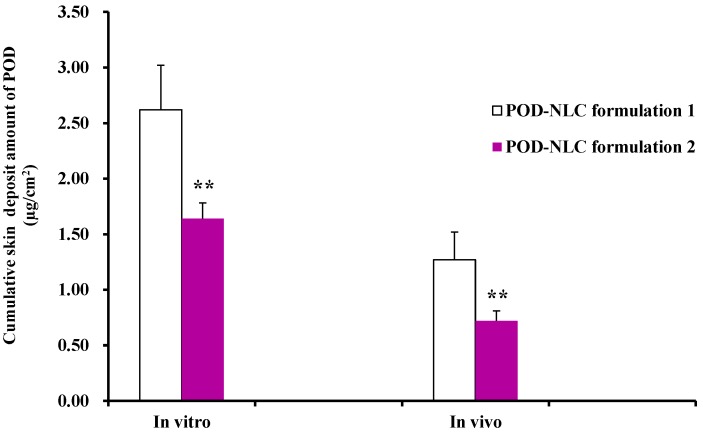
In vitro and in vivo rat skin deposit amounts of POD at 8 h after the topical treatment of POD-NLC _formulation 1_ or POD-NLC _formulation 2_. Note: “**” represents *p* ˂ 0.01 compared with POD-NLC _formulation 1_.

**Figure 5:**
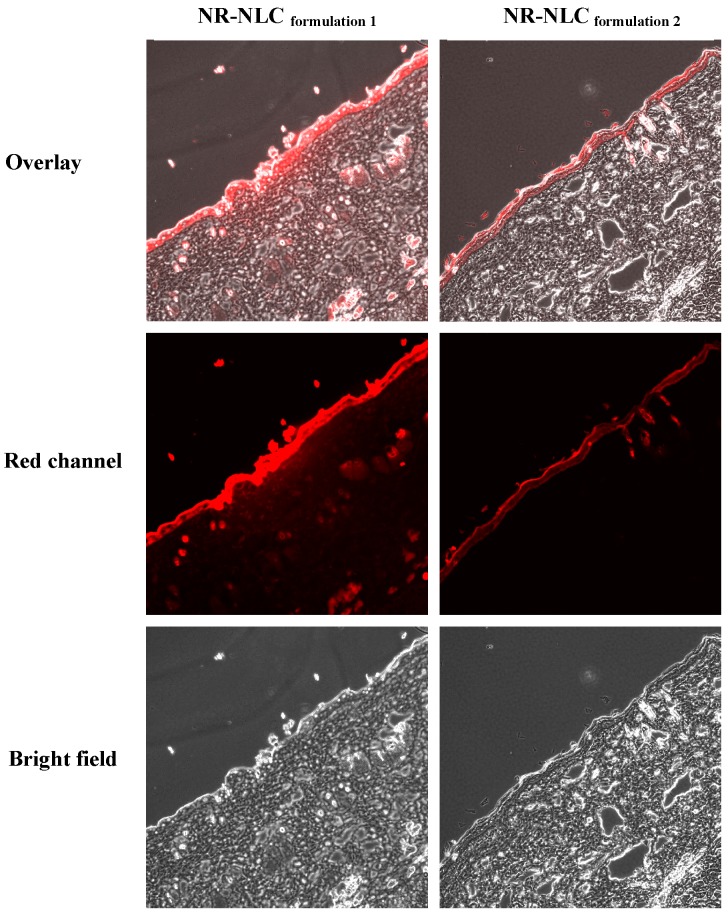
Confocal laser scanning microscopy (CLSM) images of vertical slices (10 μm) of rat skin, 4 h after the administration of Nile red-loaded NLC (NR-NLC) _formulation 1_ (109.7 nm) and NR-NLC _formulation 2_.

**Figure 6:**
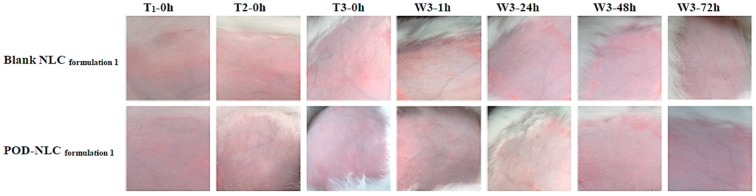
Images of intact rabbit skin before and after three topical administrations of POD-NLC _formulation 1_ or blank NLC _formulation 1_. Notes: T_1_-0 h, T_2_-0 h and T_3_-0 h represent immediately before the first, second, and third times of administration; W3-1 h, W3-24 h, W3-48 h, and W3-72 h represent 1, 24, 48, and 72 h after washing off the residual remnants of the formulation following the last administration.

## References

[1] Zhao J., Piao X., Shi X., Si A., Zhang Y., Feng N. (2016). Podophyllotoxin-Loaded Nanostructured Lipid Carriers for Skin Targeting: In Vitro and In Vivo Studies. Molecules.

